# Selective Uropathogenic *E. coli* Detection Using Crossed Surface-Relief Gratings

**DOI:** 10.3390/s18113634

**Published:** 2018-10-26

**Authors:** Srijit Nair, Juan Gomez-Cruz, Ángel Manjarrez-Hernandez, Gabriel Ascanio, Ribal Georges Sabat, Carlos Escobedo

**Affiliations:** 1 Department of Chemical Engineering, Queen’s University, Kingston, ON K7L 3N6, Canada; srijit.nair@queensu.ca (S.N.); juanmanu04@gmail.com (J.G.-C.); 2 Instituto de Ciencias Aplicadas y Desarrollo Tecnológico (ICAT), Universidad Nacional Autónoma de México (UNAM), Ciudad de México 04510, Mexico; gabriel.ascanio@ccadet.unam.mx; 3 Departamento de Salud Pública, Facultad de Medicina, Universidad Nacional Autónoma de México (UNAM), Ciudad de México 04510, Mexico; hangel@unam.mx; 4 Unidad Periférica de Patogénesis Bacteriana en Hospital General Dr. Manuel Gea González, Ciudad de México 14080, Mexico; 5 Department of Physics and Space Science, Royal Military College of Canada, Kingston, ON K7K 7B4, Canada

**Keywords:** surface plasmon resonance, urinary tract infection, surface-relief gratings, crossed surface-relief gratings, nanoplasmonics, biosensing, uropathogenic *E. coli*

## Abstract

Urinary tract infections (UTIs) are one of the major burdens on public healthcare worldwide. One of the primary causes of UTIs is the invasion of the urinary tract by uropathogenic *Escherichia coli* (UPEC). Improper treatment of bacterial infections like UTIs with broad-spectrum antibiotics has contributed to the rise of antimicrobial resistance, necessitating the development of an inexpensive, rapid and accurate detection of UPEC. Here, we present real-time, selective and label-free detection of UPEC using crossed surface-relief gratings (CSRGs) as nanometallic sensors incorporated into an optical sensing platform. CSRGs enable real-time sensing due to their unique surface plasmon resonance (SPR)-based light energy exchange, resulting in detection of a very-narrow-bandwidth SPR signal after the elimination of residual incident light. The platform’s sensing ability is experimentally demonstrated by the detection of bulk refractive index (RI) changes, with a bulk sensitivity of 382.2 nm/RIU and a resolution in the order of 10^−6^ RIU. We also demonstrate, for the first time, CSRG-based real-time selective capture and detection of UPEC in phosphate-buffered saline (PBS) solution, in clinically relevant concentrations, as opposed to other UTI-causing Gram-negative bacteria. The platform’s detection limit is calculated to be 10^5^ CFU/mL (concentration on par with the clinical threshold for UTI diagnosis), with a dynamic range spanning four orders of magnitude. This work paves the way for the development of inexpensive point-of-care diagnosis devices focusing on effective treatment of UTIs, which are a burden on public healthcare due to the rise in the number of cases and their recurrences in the recent past.

## 1. Introduction

Urinary tract infections (UTIs) are one of the most common bacterial infections worldwide. These infections are associated with escalating healthcare costs, with an estimated 10.5 million hospital visits in the United States alone in 2007, resulting in a direct and nondirect healthcare expenditure of over $2 billion [1,2]. In over 80% of cases of UTIs, the primary culprit is uropathogenic *Escherichia coli* (UPEC), which is also a major cause of many community- and healthcare-associated diseases [3,4]. The immune system controls the susceptibility of UTIs in humans and, depending on the individual’s immunity, UPECs may invade the epithelial cell lining along the urinary tract, where they grow and multiply, invading eventually other sites via the bloodstream [5,6]. UPEC detection in laboratories usually involves biochemical assays like nitrite and/or esterase tests using serological techniques, which suffer from a high probability of false-positive results [7]. At the same time, improper drug administration increases the risk of developing antibiotic-resistant bacteria [4]. Other detection techniques involve established urine culture analysis, which is time consuming (3 to 7 days), laborious and requires specialized laboratory technicians to perform [8]. Recently, genome-based detection techniques employing polymerase chain reaction (PCR) have brought the timeline of detection to a few hours [8]. These techniques, however, require highly specialized personnel to extract the genomic material for signal amplification, through tedious pretreatment methods such as cell lysis [9] and electrophoresis [10], increasing the overall cost dramatically, and thus limiting their applicability. For these reasons, the development of simple, cost-effective and time-saving devices for healthcare applications is highly sought after by the scientific community [11]. In this context, there is a timely opportunity for new healthcare diagnosis technologies to be paired with or integrated into portable electronics, which have flooded the consumer electronics market over the past few years [12]. Personal devices, such as smartphones, which have become omnipresent in recent times, provide a powerful tool for development of fully integrated point-of-care (POC) devices as diagnostic platforms [13,14,15]. With the advancement in fields of microfluidics, immunology, colorimetry, electrochemistry, light scattering-based approaches, surface plasmon resonance (SPR) and so on, researchers have developed on-site POC devices utilizing smartphone-based diagnostic platforms [16,17,18,19].

Nanoplasmonic sensors supporting SPR have been employed in various biosensing applications in the past [20,21,22,23,24,25,26]. In terms of the design of POC devices, metallic nanostructures such as surface-relief gratings (SRGs) offer several key advantages including very small footprint, portability and compatibility with collinear optics, providing easiness of integration with other microsystems [27,28]. Since surface plasmons (SPs) in SRGs can be precisely tuned by controlling the grating fabrication parameters, such as the depth and pitch, this provides a unique avenue for the development of biomedical devices at low operational and fabrication cost [29,30].

Recently, crossed surface-relief gratings (CSRGs) have been proven to be low-cost nanoplasmonic biosensors with much-improved sensing abilities compared to traditional SRGs [23]. SPs in SRGs are excited when incident light beam polarization is oriented along the grating vector, leading to the excitation of a wavelength-specific SP on a metal-coated grating [31]. This SP is normally observed as an enhanced transmission at the SPR-specific wavelength for polychromatic incident light depending not only on the light polarization, but also on the light incidence angle, the grating pitch and the refractive indices of the dielectric and the metal. CSRGs provide a different approach in SPR-based biosensing since they consist of orthogonally superimposed SRGs, allowing SPR excitation in two perpendicular light polarizations. When an incident light is polarized along the grating vector of one SRG, plasmons are excited at the metal–dielectric interface and an energy exchange takes place where the SPR resonant light is then re-radiated by the second grating in the orthogonal light polarization compared to the incident light [32]. Due to this unique phenomenon, when a broadband polychromatic light is incident on a CSRG placed between two crossed polarizers, only a narrow bandwidth corresponding to the SPR signal is transmitted, thus eliminating the rest of the incident light. Compared to other metallic nanostructures, sensing with this technique requires virtually no post-acquisition data processing, since the SPR signal is measured directly.

In this work, we present a fully integrated UPEC detection platform developed from off-the-shelf, low-cost optical components, employing CSRGs as optical sensors, with an envelope of 62.5 cm^3^ (2.5 cm × 2.5 cm × 10 cm). The platform consists of inexpensive smartphone-analogous white LED, dichroic polarizers and a portable USB spectrometer, making it suitable for point-of-care and other applications requiring portability. The sensitivity of the platform is 382.2 nm/RIU, based on bulk refractive index change tests. The platform was tested for the label-free detection of UPEC in real time, and the selectivity of the platform for UPEC was further demonstrated by performing the same experimental assays with other Gram-negative, UTI-causing bacteria. This demonstrates the potential of the platform for real-world applications and represents the first demonstration of CSRG-based UPEC detection.

## 2. Materials and Methods

### 2.1. Azo-Glass Film

Azo-glass (DR1-glass (3 wt %) solution in dichloromethane) was prepared according to the steps described elsewhere [33]. The solution was mechanically shaken for 1 h, then filtered via a 0.45 µm syringe filter (EMD Millipore, Merck KGaA, Darmstadt, Germany). Approximately 500 µL of the azo-glass solution was spin-coated on a soda lime glass slide with dimensions of 2.5 cm × 2.5 cm. Spin-coating was performed utilizing a Headway Research spin-coater (1000 rpm, 20 s), resulting in ~200-nm-thick films. The thickness of the azo-glass was measured with a Sloan Dektak II surface profiler (Veeco Instruments Inc., Plainview, NY, USA). Spin-coated substrates were later dried in a Yamato ADP-21 oven at 95 °C for 1 h, to evaporate any remaining solvent.

### 2.2. CSRG Fabrication

Orthogonally superimposed SRGs were fabricated as per the steps mentioned in [23,32]. A solid-state diode-pumped laser (Coherent Inc., Santa Clara, CA, USA, Verdi V5, λ = 532 nm) with an irradiance of 382 mW/cm^2^ in conjugation with a Lloyd mirror setup was used to generate a sinusoidal interference pattern on the azo-glass-coated substrates. This holographic exposure was achieved by two interfering beams, one inciting directly from the laser, and one reflected upon the 3 × 3 cm^2^ Lloyd mirror placed orthogonally to the sample. The resulting sinusoidal pattern, which was set to achieve 450 nm periodicity, was imprinted on the azo-glass substrate as SRGs and the area of the SRGs was controlled by a variable iris placed before the sample. With an opening of 1 cm in diameter, a grating area of 0.39 cm^2^ was achieved. After the first inscription to generate the SRG structure, the substrate was rotated by 90° and exposed to the laser interference pattern again, to generate two superimposed CSRGs with an identical pitch of 450. Subsequently, a 60-nm-thick Au film was deposited using a Bal-Tec SCD 050 sputter coater (I = 50 mA, t = 150 s), resulting in Au–CSRGs used in this work.

### 2.3. Experimental Setup

A 3D-printed custom-made assembly consisting of a 3.5 V, 20 mA, white LED (LED-w5h-ac-h110, SiLed, Mexico) used in conjugation with a plano-convex lens (7.9 mm diameter, 8 mm focal length, Edmund Optics Inc., NJ, USA) functioned as the light source. A holder was 3D-printed, using a Miicraft+ (Miicraft, Hsinchu, Taiwan) 3D printer, in order to position and collimate the LED light vertically on the sensing substrate. A horizontal polarizer (TECHSPEC^®^ Wire Grid Polarizing Film, Edmund Optics Inc., Barrington, NJ, USA) was placed directly underneath the 3D-printed assembly, accompanied by a variable iris to control the spot size of the light incident on the CSRGs. A custom sample holder, mounting the CSRGs, was positioned directly beneath the iris and a second polarizer in vertical orientation was placed after the sample to eliminate residual light after the SPR conversion. The fiber optic probe from a UV-vis spectrometer (USB 2000+, Ocean Optics, Largo, FL, USA) was positioned underneath the horizontal polarizer for maximum signal capture. All the components were arranged in a collinear arrangement atop a vertical optical rail.

### 2.4. Bacteria Culture

Bacteria *E. coli* O6:H1 (strain CFT073/ATCC 700928/UPEC), *Klebsiella pneumonia*, *Pseudomonas aeruginosa*, and *Proteus mirabilis* were routinely grown at 37 °C in Luria-Bertani (LB) medium. Overnight cultures resulted in bacterial concentration of 10^9^ colony-forming units (CFU)/mL.

### 2.5. Antibody Production

Polyclonal rabbit antiserum to *E. coli* was prepared by immunization with cell envelopes. Strain CFT073 was grown overnight at 37 °C in M9 defined culture medium (42 mM Na_2_HPO_4_, 22 mM KH_2_PO_4_, 9 mM NaCl, 18 mM NH_4_Cl, 1 mM MgSO_4_, 0.1 mM CaCl_2_ and 0.2% (w/v) glucose) [34], supplemented with 0.5 g/L of Peptone. The bacterial pellet was resuspended in 50 mM Tris-HCl, 5 mM EDTA (pH 7.5) containing protease inhibitor cocktail (Roche, Basel, Switzerland) and disrupted by ultrasonication with two 40 s pulses at low-power output, each followed by a 2-min pause, using a high-intensity ultrasonic processor (50-Watt Model, Sonics Materials Inc., Danbury, CT, USA); unbroken cells were removed by centrifugation (12,000× *g* for 10 min, 4 °C). Cell envelopes were collected by ultracentrifugation (50,000× *g* for 30 min, 4 °C) and dissolved in PBS (pH 7.4). The envelope solution was injected subcutaneously (in multiple sites, 0.5 mg without any adjuvant) into rabbits. The animals were boosted 3 and 6 weeks later with the same membrane solution. Blood was collected from the central auricular vein of each ear 15 days later.

### 2.6. Bulk RI Sensing Experiments

A thin polydimethylsiloxane-siloxane (PDMS, Sylgard 184, Dow Corning, Midland, MI, USA) layer, of approximately 2 mm in thickness, was prepared by methods described elsewhere [35]. An 8 mm × 8 mm hole was cut onto a 2 cm × 2 cm piece of the prepared thin PDMS layer, and it was placed on top of the crossed pattern region of the CSRGs. Sucrose solutions in deionized water with different weight percentages (5%, 10%, 15% and 20%) were prepared to be used as test solutions for bulk refractive index sensing. The refractive indices of the solutions were measured utilizing an Abbe refractometer (Shanghai Optical Instruments Co. Ltd., Shanghai, China).

### 2.7. Bacterial Detection

The surface of the CSRGs was cleaned and prepared by rinsing with 10% acetone and deionized water before the bacterial immobilization. Subsequently, the test solution was dispensed in the PDMS well to be hosted over the CSRG surface. Next, a baseline signal was acquired after introducing phosphate-buffered saline (PBS) solution into the PDMS well. The SPR peak acquired corresponding to the PBS transmission signal was used to calibrate the SPR peak-shift observed in the later part of the experiment. Next, UPEC-specific antibody solution was introduced onto the surface of CSRGs, and the corresponding SPR signal was recorded for 15 min. Thereafter, the sample was rinsed with PBS solution to discard any antibody not attached to the surface. The bacterial suspensions in PBS were inserted, recording the corresponding SPR peak shifts for 15 min. For UPEC, the transmission spectra from the CSRGs were acquired in real time, every 2 min. For other bacteria, the transmission spectra were obtained every 4 min.

## 3. Results and Discussion

### 3.1. Crossed Surface-Relief Gratings

**Figure 1:**
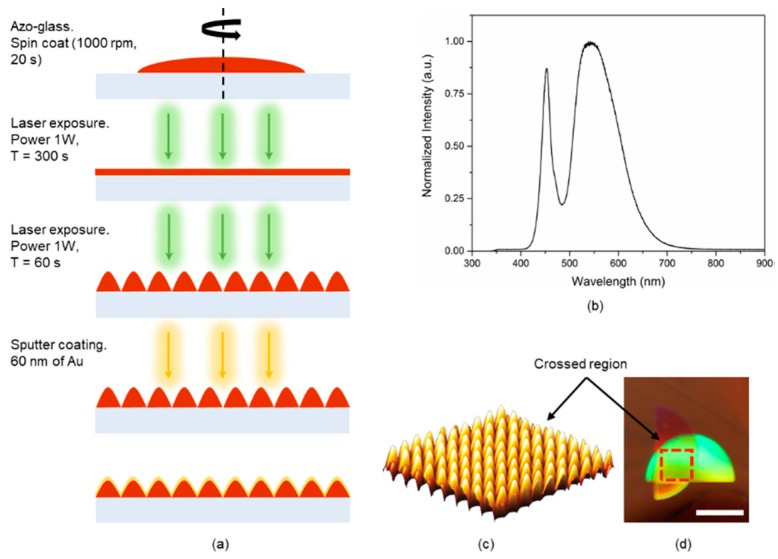
(**a**) Schematics of the fabrication procedure for Crossed Surface Relief Gratings (CSRGs). (**b**) Normalized spectra for white LED used in this work. (**c**) Atomic force microscopy (AFM) scan of 4 µm × 4 µm crossed region showing the orthogonally superimposed SRGs. (**d**) Actual image of the fabricated CSRGs with the crossed region marked with red box. White scale bar corresponds to 1 cm.

Figure 1a shows the CSRG nanofabrication procedure. The nanostructures were fabricated on substrates coated with azo-glass through a laser inscription technique. The SPR signal associated with CSRGs is attributed to the SPR energy conversion occurring between the individual SRGs. For normally incident light, the SPR excitation wavelength (λ_SPR_) depends on the pitch (Λ) of the gratings, and the propagation characteristics of the media such as the dielectric permittivities of the metallic film, ε_m_, and the surrounding dielectric medium, ε_d_. The intensity or signal strength of the standing-wave surface plasmon is dependent on the depth of the gratings. For CSRGs, the SPR energy conversion occurs at λ_SPR_ when the light momentum is phase-matched between the diffracted incident light and the surface plasmon. Thus, for normal light incidence, λ_SPR_ can be represented as:

λ_SPR_ = ηΛ [ε_m_/(η^2^ + ε_m_)]^1/2^, (1)

where η is the refractive index of the surrounding dielectric medium, and η = (ε_d_)^1/2^. From Equation (1), it can be inferred that an increase in η would result in an increase in λ_SPR_. The thickness of the azo-glass layer also plays an important role in transmission spectroscopy since an azo-glass film absorbs light below 550 nm. Thus, a thick azo-glass film may result in a decreased surface plasmon signal in transmission. On the other hand, a very thin coating of azo-glass may result in shallow gratings, greatly reducing the intensity of the transmitted SPR signal. Accordingly, the thickness of azo-glass film was optimised to approximately 200 nm for all the fabricated sensors. Also, to avoid the absorbance by azo-glass, the pitch of the gratings was chosen to excite plasmons above 600 nm. Another factor that influenced the choice of pitch of the gratings was the light source. Since the system is intended for smartphone-based platforms, the white LED used in this work is analogous to a smartphone flash LED. As evident from Figure 1b, the spectra of the white LED used for this work has a maximum around 550 nm, eventually tailing-off until there is no light above 700 nm. Lastly, it must be considered that the optical platform is to be employed for water-based samples: sucrose solutions, PBS and bacterial solutions. Taking all the aforementioned factors into account, it was desirable to achieve the SPR signal in the range of 600–700 nm for the test solutions. From Equation (1), based on 450-nm pitch gratings, a theoretical λ_SPR_ of 648 nm can be calculated for water as the surrounding dielectric medium. Using this input parameter, 450-nm-pitch CSRGs were fabricated by orthogonal superimposition of individual SRGs with identical 450-nm pitch. First, a 450-nm-pitch SRG was laser-inscribed on the azo-glass substrates using a solid-state diode-pumped laser (irradiance = 382 mW/cm^2^) by direct holographic exposure for 300 s. Next, the substrate was rotated 90°, and a second SRG, superimposed on the initial grating, was laser-inscribed for 60 s. This resulted in orthogonally superimposed SRGs (i.e., CSRGs) of similar depth and diffraction efficiencies. Surface topography analysis using AFM shows the generation of CSRGs with a depth of c.a. 75 nm and desired pitch of 450 nm, as shown in Figure 1c. Subsequently, sputter deposition was performed to coat the fabricated CSRGs with 60-nm-thick Au film. Figure 1d shows the actual CSRG sensors, with the grating region showing the multi-colored diffractions. It is worth mentioning that the nanofabrication protocol, involving the azo-glass, provides a cheaper alternative to the clean-room-based techniques, and at the same time, allows nanometer precision in fabrication of gratings by controlling the fabrication parameters such as laser power, laser angle of incidence on the substrate, and time of exposure. This permits precise control over the depth and pitch of the gratings, allowing the freedom to design CSRGs based on the desired SPR signal wavelength.

### 3.2. Optical Characterization

**Figure 2:**
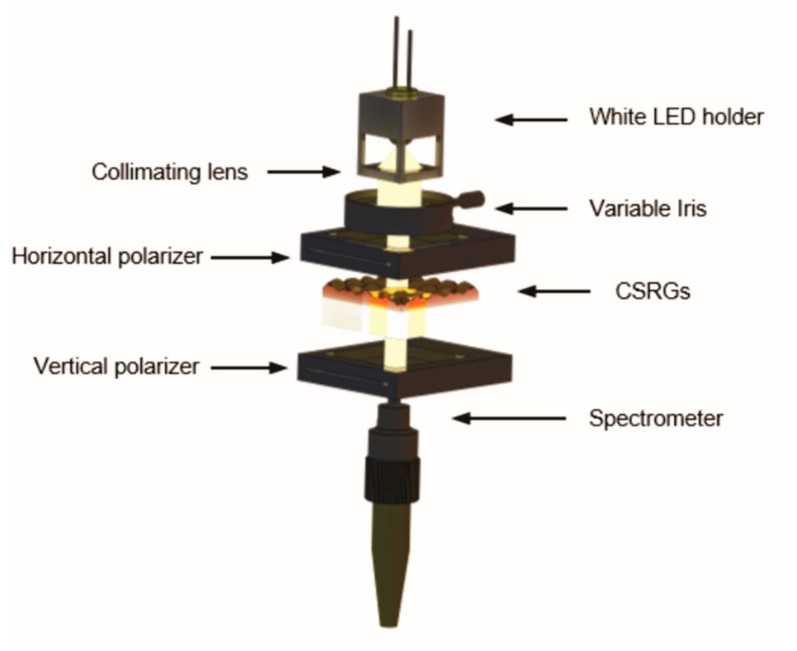
Schematic of the optical platform for transmission-based spectroscopy using CSRGs. All the elements are arranged in a collinear arrangement on a vertical rail. The light from the white LED passes through a variable iris, to control the spot diameter upon the horizontal polarizer, which is then incident on the CSRGs exciting the plasmons. The resulting out-coupled light then traverses the vertical polarizer, annulling all residual light from the white LED source, except the plasmonic signal detected by the spectrometer.

As the system is intended to be used, ultimately, as a smartphone-based platform, one of the critical goals was to reduce the footprint of the optical platform. Figure 2 shows the schematic representation of the experimental setup developed for this work. Collimated white light from the 3D-printed assembly was first directed towards the horizontally aligned polarizer. The horizontally polarized light was then incident on the metallic CSRGs. At this juncture, surface plasmons are excited at the metal–dielectric interface of the CSRGs, by the first grating having a horizontal grating vector. An SPR energy exchange then occurs between the first grating and its orthogonal component, having a vertical grating vector. This SPR energy is then re-radiated by the second grating, as explained elsewhere [23,32]. This resulting out-coupled light has a polarization orthogonal to the incident horizontally polarized light. Therefore, placing a vertical polarizer downstream from the CSRGs eliminates the entire incident light, except for the re-radiated SPR signal from the CSRGs. This unique feature allows acquisition of SPR signals without any further normalization of the transmitted light (with the transmission spectra of gold or source light).

### 3.3. Bulk Refractive Index Sensing

**Figure 3:**
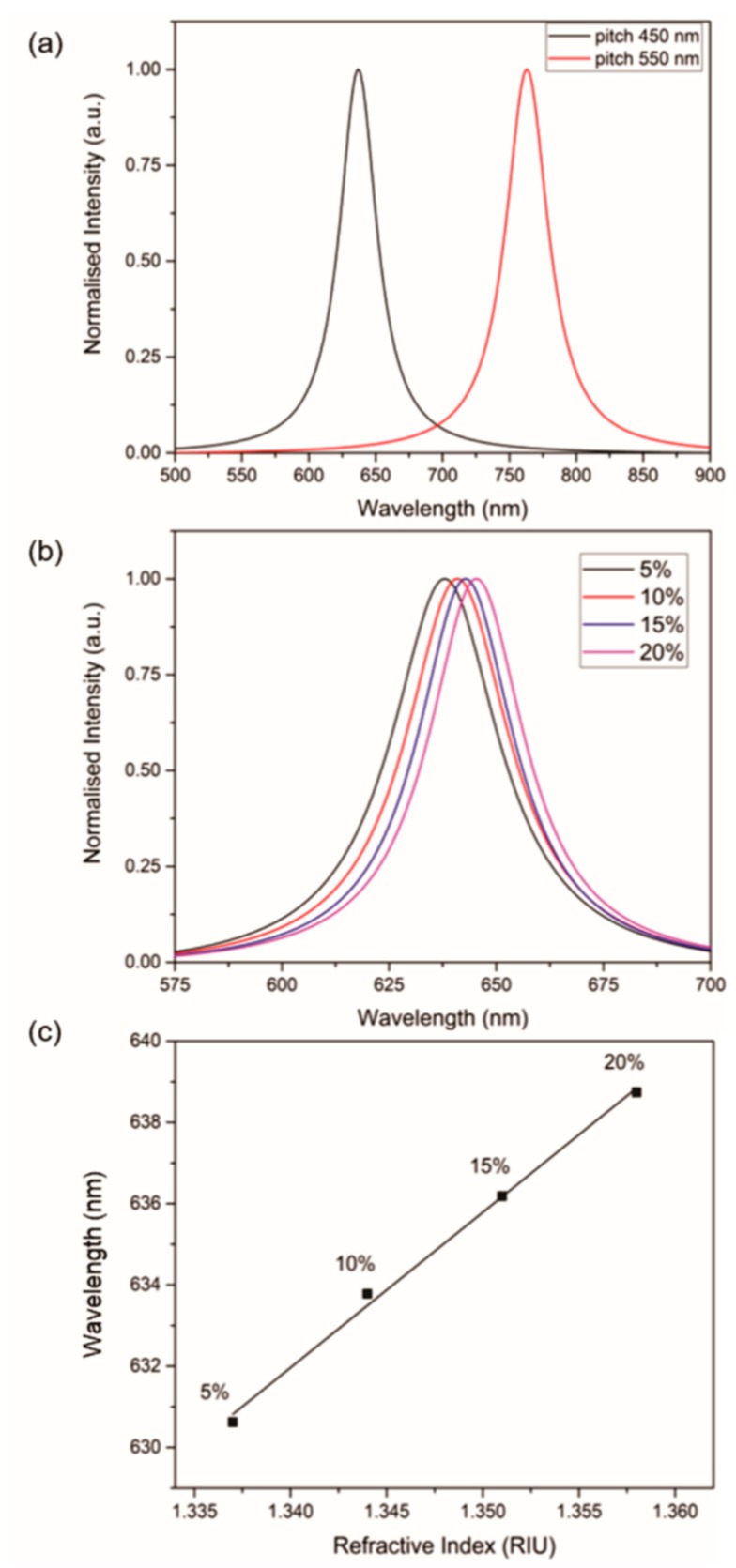
Bulk sensitivity test. (**a**) Normalized surface plasmon resonance (SPR) peaks for water acquired using two different pitch CSRGs (450 nm and 550 nm). (**b**) Normalized SPR peaks for aqueous sucrose solutions of different concentrations (5%, 10%, 15% and 20%). The SPR spectrum shifts toward red as the refractive index increases. (**c**) Wavelength (nm) vs refractive index (RIU) for each solution. The sensitivity of the platform is 382.2 nm/RIU, based on the slope of the linear fit. No error bars are indicated since the standard deviation for *N* = 3 is smaller than the size of the symbol representing the mean in the graph.

Figure 3a shows the SPR signal acquired for deionised water using two different CSRGs, illuminated by a broadband halogen lamp, with the first CSRG having equal pitches of Λ = 450 nm and the second CSRG having equal pitches of Λ = 550 nm. From Equation (1), the theoretically calculated λ_SPR_ for deionised water is 648 nm and 765 nm for Λ = 450 nm and 550 nm, respectively. Experimentally, λ_SPR_ for deionised water, calculated from the acquired spectra, was found to be 637 nm and 761 nm. The difference between the theoretical and observed λ_SPR_ is mainly due to the flat interface approximation considered when deriving Equation (1). Nonetheless, these values are sufficiently close to display the precision of the nanofabrication method in tailoring the SPR response with respect to the end-application. Next, the performance of the miniaturized setup was evaluated to detect changes in the bulk refractive index by using aqueous sucrose solutions of 5%, 10%, 15% and 20% concentration (w/v), with respective RIs of 1.337, 1.344, 1.351 and 1.357, measured using the Abbe refractometer. A thin PDMS slab (2 cm × 2 cm) with an 8 mm × 8 mm chamber was placed on the CSRGs, in order to allow liquid–metal contact. The liquid in the chamber, ~140 µL, was covered with a cover slip to eliminate any potential lensing effect. The transmitted spectrum was acquired for each solution, as per the setup described previously. Figure 3b shows the spectra, and corresponding SPR peaks, of the sucrose test solutions. The SPR spectrum shows a characteristic red-shifting, corresponding to the increase in RI, as explained earlier and as prescribed by Equation (1). The SPR signals were normalized and the total peak-shift at 80% maximum intensity was recorded. Figure 3c shows the recorded linear peak-shift associated with the increments in the refractive indices of the test solutions. The sensitivity of the platform, obtained from the linear fit of the peak-shift, was 382.2 nm/RIU, which is comparable to previously reported values of SRG-based sensors operating in transmission mode [36]. It should also be noted that the platform presented here employs off-the-shelf and inexpensive optical components, lowering the device fabrication cost considerably as compared to similar systems reported previously [23]. The resolution of our system, based on calculated sensitivity and system repeatability of 10^−3^ nm, is 10^−6^ RIU [37]. This value is particularly important since it gives information about the efficacy of our device by taking the system noise into account.

### 3.4. Bacterial Detection

**Figure 4:**
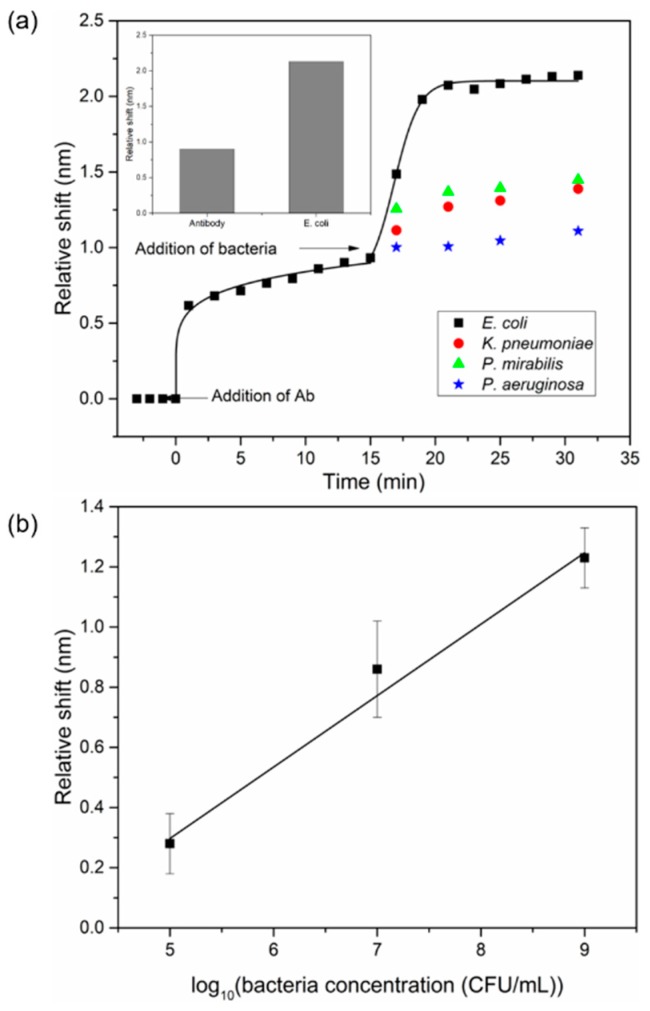
Selective uropathogenic *Escherichia coli* (UPEC) detection assay. (**a**) Real-time relative shift corresponding to capture of UPEC bacteria, and other UTI-causing Gram-negative bacteria at concentration 10^9^ CFU/mL. Inset shows the relative cumulative shift in SPR signal observed after binding of UPEC-specific antibody and UPEC. (**b**) Relative shift corresponding to different concentrations of UPEC in PBS buffer (10^5^, 10^7^, 10^9^ CFU/mL). Error bars indicate the standard deviation observed for each bacterial measurement for *N* = 3.

The utility of the sensing platform to detect UPEC was investigated. The employed schema focused on detection of intact bacteria, as opposed to genome-based sequencing techniques, which involve time-consuming steps such as DNA extraction, PCR and subsequent processing. Also, clinical UTI detection involving urine culture is laborious and involves qualified technicians and specialized facilities, resulting in delayed detection timelines. The platform presented here overcomes the drawbacks of such methods, reducing the UPEC detection time from days to minutes. UPEC-specific antibodies, prepared as described in the Methods section, immobilize the bacteria by binding to their outer membrane. Binding is facilitated by anchoring of proteins, phospholipids and oligosaccharides to the cells’ surface [38]. The surface of the CSRGs was functionalized with UPEC-specific antibodies to enable whole-bacterium detection. Figure 4a shows the relative SPR peak shift observed in real time for both antibodies (t = 0 to t = 15 min) and for detection of bacteria (t > 15 min) taking the signal for PBS as baseline (t = 0 min). The UPEC-specific antibodies were incubated on the CSRG surface for 15 min, and the transmission spectra were acquired every 2 min. The immobilization of the antibodies on the surface of CSRGs promoted an increase in the local refractive index at the metal–dielectric interface. This increase was reflected as a red-shift in the transmission spectra (i.e., SPR peak) as theorized by Equation (1). Next, UPEC solution in PBS (10^9^ CFU/mL) was added to the antibody-modified CSRG surface, and the transmission spectra were acquired every 2 min for another 15 min. The real-time displacement in the SPR spectra, due to the antibody and bacteria immobilization, is presented in Figure 4a as a function of time (black square). As evidenced by the inset in Figure 4a, the addition of antibody and bacteria resulted in a respective 0.9 nm and 2.13 nm shift, compared to the PBS baseline. Another goal of this work was to demonstrate the platform’s specificity in detection of UPEC. The selectivity of the platform was validated by performing the same experiment with other UTI-causing, Gram-negative bacteria, namely: *Klebsiella pneumonia*, *Pseudomonas aeruginosa* and *Proteus mirabilis*. After initial incubation of UPEC-specific antibody for 15 min, 140 µL of the bacterial solution in PBS (10^9^ CFU/mL) was added, and the transmission spectra were recorded for 15 min, at five-min intervals. Colored symbols (other than black) after t = 15 min represent other nonspecific bacteria. The platform was highly specific for the detection of UPEC, evident from the very small shift observed with the other Gram-negative bacteria, as shown in Figure 4a. The platform was also tested for any potential drifting by monitoring the signal over time using DI water, PBS solution, and bound streptavidin to a cysteamine/biotin complex immobilized atop the CSRG surface, under quasi-steady-state conditions as reported elsewhere [23] (details available in the Supplementary Materials). The limit of detection (LOD) of the CSRG-based platform was determined by a dose-based binding study. UPEC solutions in PBS with concentrations of 10^5^, 10^7^ and 10^9^ CFU/mL were used in this experiment. Figure 4b shows the relative shift of the plasmonic signal, detected from the antibody-functionalized CSRG surface upon addition of different concentrations of UPEC solution. Based on this study and taking into consideration the resolution of the spectrometer, we calculated the LOD of the platform to be approximately 10^5^ CFU/mL, with a dynamic range of four orders of magnitude (10^5^–10^9^ (CFU/mL)) [39,40]. This value is on par with the clinical threshold for UPEC concentrations (10^5^ CFU/mL) in UTI diagnosis [3]. The SPR peak-shifts observed in this experiment are accordant with the bacterial detection studies previously reported in the literature [41,42,43]. This experiment, notably, represents the first demonstration of CSRG-based bacterial detection.

## 4. Conclusions

This work presents the first demonstration of label-free detection of bacteria by CSRGs as a nanoplasmonic sensor. A fully integrated, miniaturized (2.5 cm × 2.5 cm × 10 cm) platform consisting of smartphone-compatible, inexpensive optical and electronic components in conjugation with CSRGs is employed for SPR-based sensing. The platform demonstrates a sensitivity of 382.2 nm/RIU, with a resolution of 10^−6^ RIU, for bulk refractive index changes. The sensitivity of the platform depends not only on the integrity and characteristics of the metallic nanostructure, but also on the optical assembly, including the quality of its components, employed for sensing. Despite the low-cost optical components used in this work, the sensitivity is still on par with similar nanoplasmonic assemblies in the literature. We employed the platform for selective detection of UPEC suspended in PBS solution, demonstrating its potential in real-world applications. The platform was able to detect UPEC capture by immobilized antibodies on the CSRG surface, at concentrations from 10^5^ to 10^9^ CFU/mL, with the whole detection being performed in 35 min as opposed to clinical detection timelines of days. The platform has a dynamic range spanning four orders of magnitude, with an experimental LOD for UPEC detection in PBS to be ~10^5^ CFU/mL, which is on par with the physiological limit for UTI diagnosis. Along with the low cost of the platform and sensor, the detection was carried out with minimal sample pretreatment as opposed to established genomic techniques, which require time-consuming assays to extract and amplify bacterial genome. In the future, detection of UPEC in complex biological matrices, like human urine, would further advance and solidify the platform’s applicability as a point-of-care device. The platform, however, had limitations in terms of detection of lower concentrations of bacteria due to the resolution of the USB spectrometer. However, this drawback can be overcome by using SPR imaging (SPRi) techniques. Imaging components such as complementary metal-oxide semiconductor (CMOS) may improve the resolution of the platform considerably. Furthermore, incorporation of microfluidic components could improve upon the functionality of the detection platform for complex applications including multiplexed sensing. Overall, the platform presented here has great potential to advance the field of smartphone-based sensing and telemedicine, with a wide range of applications.

## Supplementary Materials

The following are available online at http://www.mdpi.com/1424-8220/18/11/3634/s1, Figure S1: Relative shift in SPR peak recorded for water from t = 0 min to t = 20 min, with 30-s interval between signal acquisition. Figure S2: Relative shift in SPR peak recorded for PBS solution from t = 0 min to t = 20 min, with 30-s interval between signal acquisition. Figure S3: Relative shift in SPR peak recorded for bound streptavidin on the immobilized cysteamine/biotin complex on CSRG surface from t = 0 min to t = 20 min, with 1-min interval between signal acquisition.
