# Supplementary material for: Selective Uropathogenic E. coli Detection Using Crossed Surface-Relief Gratings
Source: Sensors (Basel). 2018 Oct 26;18(11):3634. doi: 10.3390/s18113634 (PMC6263983; doi:10.3390/s18113634)
Supplement: Supplementary file 1 [file sensors-18-03634-s001.pdf]

sensors

MDPI

Article

# Selective Uropathogenic *E. coli* detection using crossed surface relief gratings

**Srijit Nair [1,§], Juan Gomez-Cruz [1,2,§], Ángel Manjarrez-Hernandez [3,4], Gabriel Ascanio [2], Ribal Georges Sabat [5,*] and Carlos Escobedo [1,*]**

[1] Department of Chemical Engineering, Queen's University, Kingston, ON, Canada K7L 3N6
[2] Centro de Ciencias Aplicadas y Desarrollo Tecnológico (CCADET), Universidad Nacional Autónoma de México (UNAM), Ciudad de México 04510, México
[3] Departamento de Salud Pública, Facultad de Medicina, Universidad Nacional Autónoma de México (UNAM), Ciudad de México 04510, México
[4] Unidad Periférica de Patogénesis Bacteriana en Hospital General Dr. Manuel Gea González, Ciudad de México 14080, México
[5] Department of Physics and Space Science, Royal Military College of Canada, Kingston, Ontario K7K 7B4, Canada;

§ Equal contribution

* Correspondence: sabat@rmc.ca (R.G.S.); ce32@queensu.ca (C.E.); Tel.: +1-613-541-6000 ext. 6721(R.G.S.); Tel.: +1-613-533-3095 (C.E.)

## Supplementary Materials

*SPR peak drift experiments*

Experiments were performed to determine any potential drift of the acquired signal when using DI water and PBS solutions as test samples. A thin PDMS slab (2 cm × 2 cm) with an 8 mm × 8 mm chamber was placed on the CSRGs, in order to allow liquid-metal contact. The liquid in the chamber (~140 µL) was covered with a cover slip to eliminate any potential lensing effects. Transmitted signal from the CSRGs was acquired for 20 min, with 30-s intervals between each acquisition. As shown in Figures S1 and S2, no considerable drift in the SPR signal was observed over the 20 min, for both DI water and PBS solution. A cysteamine/biotin/streptavidin assay, as reported elsewhere [1], was also performed in order to tabulate the potential drift associated with respect to binding of analytes and biomolecules on the CSRGs surface, akin to the bacterial detection presented in the article. A streptavidin solution (800 nM) was incubated atop a cysteamine/biotin complex immobilized on the surface of the CSRGs and the system was allowed to reach quasi-steady state. The signal from the system was acquired for 20 min, with 1-min interval between each acquisition, as shown in Figure S3. The signal was quite stable over the lapse of the experiment and no significant drift was observed.

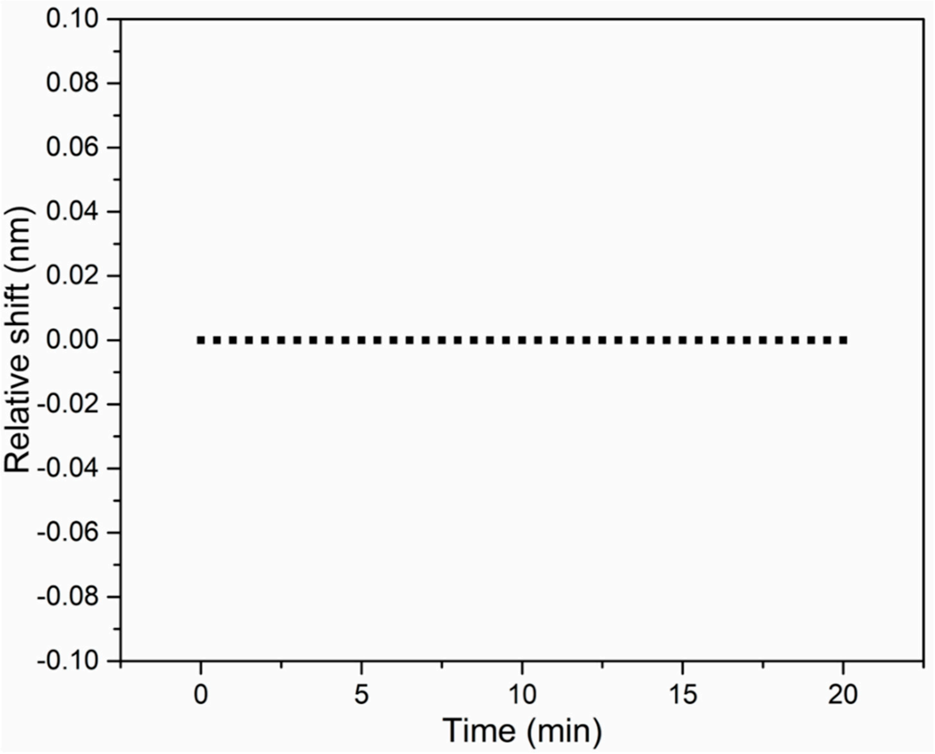


**Figure S1.** Relative shift in SPR peak recorded for water from t = 0 min to t = 20 min, with 30-s interval between signal acquisition.

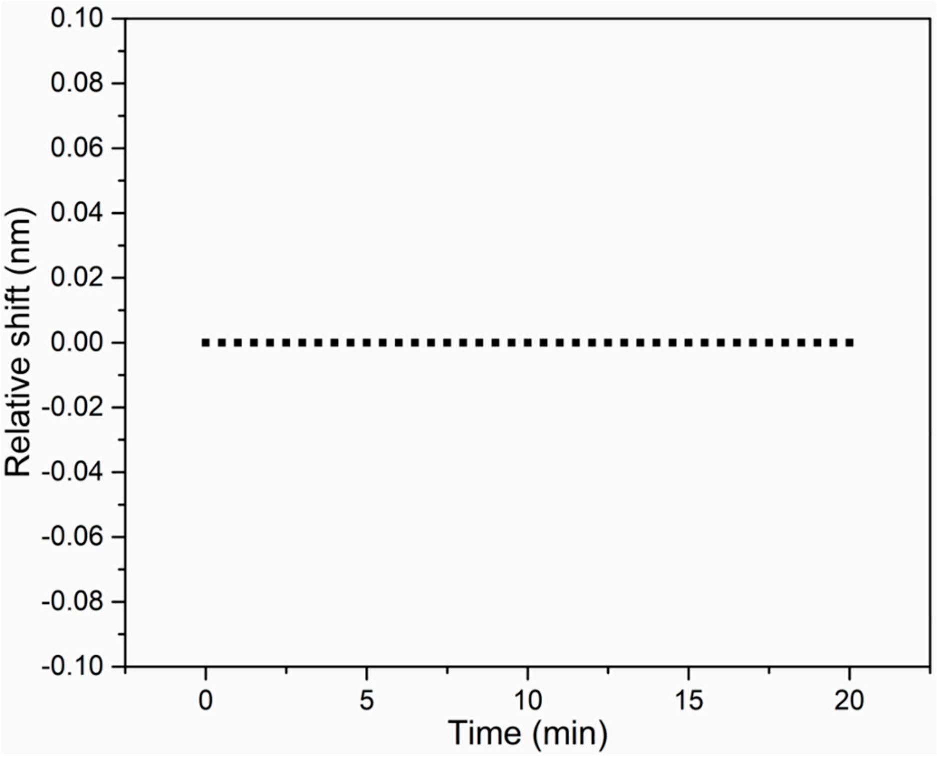


**Figure S2.** Relative shift in SPR peak recorded for PBS solution from t = 0 min to t = 20 min, with 30-s interval between signal acquisition.

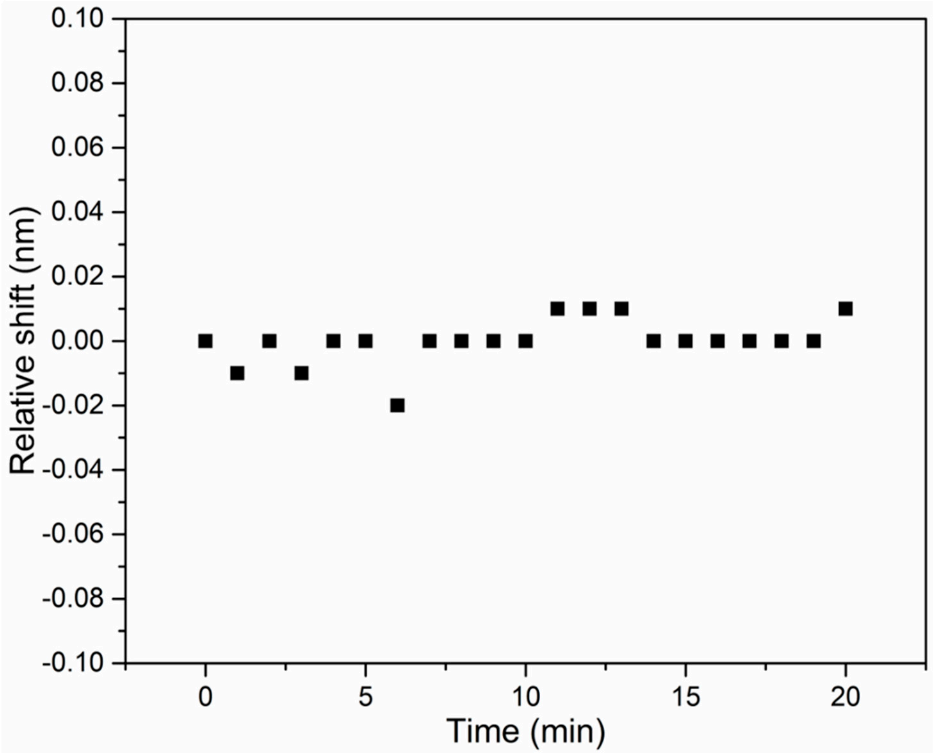


**Figure S3.** Relative shift in SPR peak recorded for bound streptavidin on the immobilized cysteamine/biotin complex on CSRGs surface from t = 0 min to t = 20 min, with 1-min interval between signal acquisition.

## Reference

1. Nair, S.; Escobedo, C.; Sabat, R. G. Crossed Surface Relief Gratings as Nanoplasmonic Biosensors. *ACS Sensors* **2017**, *2*, 379–385, doi:10.1021/acssensors.6b00696.
